# Onco-Preventive and Chemo-Protective Effects of Apple Bioactive Compounds

**DOI:** 10.3390/nu13114025

**Published:** 2021-11-11

**Authors:** Linda Nezbedova, Tony McGhie, Mark Christensen, Julian Heyes, Noha Ahmed Nasef, Sunali Mehta

**Affiliations:** 1 School of Food and Advanced Technology, Massey University, Palmerston North 4442, New Zealand; l.nezbedova1@massey.ac.nz (L.N.); j.a.heyes@massey.ac.nz (J.H.); 2 Riddet Institute, Massey University, Palmerston North 4442, New Zealand; n.nasef@massey.ac.nz; 3 The New Zealand Institute for Plant and Food Research Limited, Palmerston North 4442, New Zealand; tony.mcghie@plantandfood.co.nz; 4 Heritage Food Crops Research Trust, Whanganui 4501, New Zealand; mark@heritagefoodcrops.co.nz; 5 Pathology Department, Dunedin School of Medicine, University of Otago, Dunedin 9054, New Zealand; 6 Maurice Wilkins Centre for Biodiscovery, University of Otago, Dunedin 9054, New Zealand

**Keywords:** fruit, apple, phytochemicals, cancer, chemoprevention, antioxidants, phenolics, triterpenoids

## Abstract

Cancer is one of the leading causes of death globally. Epidemiological studies have strongly linked a diet high in fruits to a lower incidence of cancer. Furthermore, extensive research shows that secondary plant metabolites known as phytochemicals, which are commonly found in fruits, have onco-preventive and chemo-protective effects. Apple is a commonly consumed fruit worldwide that is available all year round and is a rich source of phytochemicals. In this review, we summarize the association of apple consumption with cancer incidence based on findings from epidemiological and cohort studies. We further provide a comprehensive review of the main phytochemical patterns observed in apples and their bioavailability after consumption. Finally, we report on the latest findings from in vitro and in vivo studies highlighting some of the key molecular mechanisms targeted by apple phytochemicals in relation to inhibiting multiple ‘hallmarks of cancer’ that are important in the progression of cancer.

## 1. Introduction

Chronic diseases including cancer continue to remain a public health burden globally [1,2,3,4,5]. In 2020, cancer was the second leading chronic illness following cardiovascular disease, with an estimate of 19 million new cases and accounting for 10 million deaths per year, globally [6,7]. Data from GLOBOCAN [7] show that cancers of the breast are the most commonly diagnosed followed by cancers of the lung, colorectal, and prostate. 

To reduce cancer’s global health burden, it is necessary to promote both cancer treatment and cancer prevention. There is growing evidence that phytochemicals found in vegetables and fruits play a major role in cancer aetiology. Furthermore, diet and simple dietary changes incorporating fruits and vegetables can influence the risk of cancer [8]. Apples are an example of commonly available fruits worldwide that are a rich source of phytochemicals. There is a milieu of studies around the health benefits of apples including onco-preventive effects. However, translating this information into an appropriate intervention requires understanding of how the different components of apples contribute to their health benefit. These components include differences in the phytochemical concentration within the skin vs. flesh of the apple, the impact of the apple food matrix, and absorption and bioavailability of apple phytochemicals. In this review, we provide detailed insight into selected dietary phytochemicals found in apples, their onco-protective role in cancer, and their effect on different pathways implicated in cancer development and progression. This review collates details from different studies related to apples and apple phytochemicals in order to provide a more holistic understanding around the effects of apple consumption on cancer. We also highlight the gaps in the literature to promote more relevant studies in this field including clinical trials.

## 2. Cancer

Cancer is a group of heterogeneous diseases that can occur in multiple parts of the body [9]. Triggers for cancer are complex and include genetic predispositions, environment, and lifestyle [10,11]. In this section, we briefly describe the different steps involved in cancer development (carcinogenesis) to better understand the pathways targeted by different phytochemicals described in subsequent sections of this review.

Carcinogenesis is a multi-step process involving initiation, promotion, and progression of cancer [12,13,14]. Initiation is the first irreversible step of carcinogenesis and refers to the genetic and epigenetic alterations in somatic cells mainly involving proto-oncogenes such as RAS, c-Myc, and tumor suppressor genes such as Rb and p53 [15,16,17,18,19]. Promotion is the second phase of carcinogenesis where non-mutagenic promoting agents result in reversible changes in the genome, giving cells the ability to proliferate uncontrollably and expand [14,20]. Growth factors such as epidermal growth factors (EGF), hormones including estrogen, and external factors such as chemical compounds from diet are examples of agents promoting these non-mutagenic events within the cell [21,22]. Progression represents a later stage of tumor development and is characterized by accumulation of multiple genetic changes, including increased mutational load, number, and arrangement of chromosomes and epigenetic changes [23]. Continuous accumulation of genomic changes within the cells allows them to acquire multiple ‘hallmarks of cancer’ including uncontrolled growth, ability to resist cell death, alter metabolism, evade the immune system, and invade and spread to other tissues and organs (reviewed in detail [24]). 

## 3. Importance of Phytochemicals from Diet in Management of Cancer

Diet can influence cancer development in both positive and negative ways. It is estimated that a healthy lifestyle and healthy dietary practices could help lower incidence of all cancers by 30-40% [25]. Furthermore, a diet rich in vegetables, fruits, whole grains, dietary fiber, omega-3 fatty acids, and certain micronutrients (e.g., vitamins and calcium) protects against some cancers [26,27,28]. In contrast, diets rich in meat, processed foods, fried foods, and smoked foods can increase the risk of developing some cancers [29,30,31]. 

For a healthy diet, the World Health Organization (WHO) recommends the consumption of a minimum of five portions or 400 g of fruit and vegetables per day, of which two portions should constitute fruit [32,33]. Studies have shown differences in fruit consumption across the world based on the socioeconomic status, sex, and geographic location. From these studies, cancer incidence was estimated to decrease by 14% with consumption of 550–600 g of fruit and vegetable per day, which is greater than the current recommendations made by WHO [34,35]. These results suggest that a healthy diet such as one high in fruits and vegetables has protective effects against different types of cancer.

Protective effects of fruit against cancer are related to their high content of bioactive compounds (phytochemicals). Phytochemicals are secondary metabolites from plants responsible for the taste, color, and aroma of fruit. Research suggests that phytochemicals are beneficial in preventing and treating oxidative damage and inflammation, which are important risk factors in cancer development [36,37,38,39]. Moreover, phytochemicals have significant onco-preventive and chemo-protective effects, and this has been reviewed extensively elsewhere [38,40,41,42,43,44,45]. 

One fruit that is a rich and important source of bioactive phytochemicals in Western diets is the apple [46,47,48,49]. Apples are globally consumed due to their year-round availability, their cultivar diversity, low price, and easy storage [46,50]. The subsequent sections of this review will focus specifically on the onco-preventive and chemo-protective properties of phytochemicals found in apples.

## 4. Apple Phytochemical Profile and Bioavailability

To better understand the health benefits of apples, in this section we provide a comprehensive review of the main phytochemical patterns observed in apples and their potential health benefits depending on variety and the consumed part of the fruit (skin/peel versus the flesh of the apple). 

**Figure 1:**
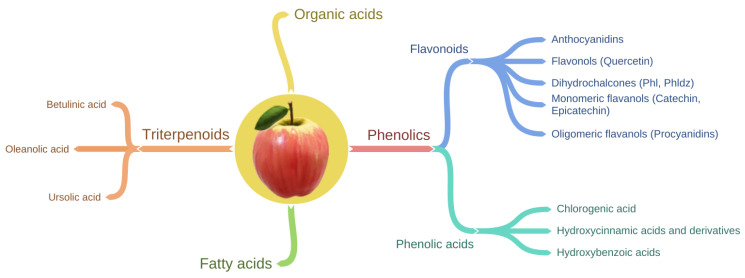
Schematic showing the classification of phytochemicals present in apple.

Apples contain a wide variety of phytochemicals, including triterpenoids, organic acids, fatty acids, and apple phenolic compounds (Figure 1) [51]. Triterpenoids are components mainly of apple waxes [52]. The main triterpenoids found in apples are oleanolic, betulinic, and ursolic acid and their derivatives such as maslinic, corosolic, euscaphic, pomaceic, and pomolic acids [53].

The most well-studied group of apple phytochemicals for their health benefits are phenolic compounds [54]. Studies show that apples are an important source of phenolic compounds in our diet contributing to 22% of phenolic intake [55,56]. Most of the phenolic compounds in the fruit are usually present in the conjugated form such as glycosides or esterified carboxylic acids. However, compared to other fruit, apples contain more of the readily bioavailable free forms of phenolic compounds [57,58,59]. For instance, the ‘Red Delicious’ apple had the highest level of free forms of phenolic compounds compared to pear, plum, kiwifruit, and peach [60]. 

Phenolic compounds in apple can be sub-divided into two main groups (Table 1, Figure 1) known as flavonoids and phenolic acids. Flavonoids can be further divided into four structural subclasses including anthocyanidins, flavonols, dihydrochalcones, and flavan-3-ols (flavanols) which can exist in the monomeric and oligomeric form [49,61] (Table 1). Phenolic acids include chlorogenic acid, hydroxycinnamic acid, and hydroxybenzoic acid [49,61]. In general, chlorogenic acid and monomeric and polymeric flavanols are the major phenolic compounds, whereas anthocyanins and dihydrochalcones are minor phenolic compounds of apples [62]. Moreover, anthocyanidins are responsible for the apple redness [63,64]. Therefore, anthocyanidins are abundant in the apple cultivars with red skin (e.g., ‘Red Delicious’) and are either present in low concentrations or absent in green skinned apple cultivars (e.g., ‘Granny Smith’) [64,65].

**Figure 2:**
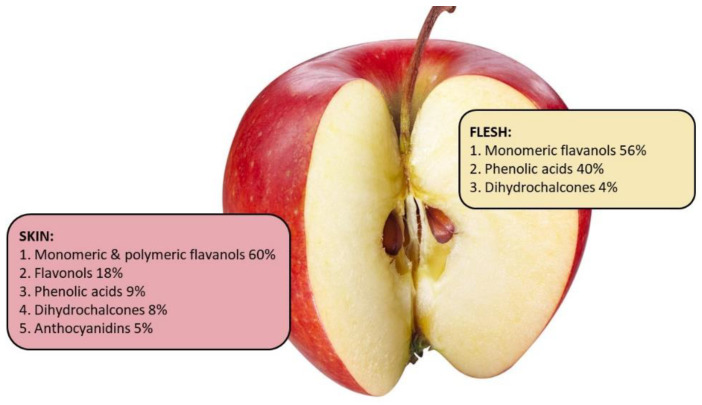
Phenolics distribution pattern in the peel and flesh of an average apple based on the data from Lata, Trampczynska, and Paczesna [71], Tsao et al. [78], Lata [79], and McGhie et al. [62].

There are differences in the distribution and type of phenolic compounds within various parts of an apple such as peel, flesh, core, and seeds. The phenolic compounds distribution and concentration in the peel and flesh of apples vary greatly due to genetic diversity, maturity stage, growing conditions and geographical location, harvest, and storage conditions [59,66,67,68,69,70]. However, studies have highlighted that there is a similar phenolics distribution pattern for most apple cultivars (Figure 2).

In general, the apple peel contains about 2–4 times higher concentration of phenolic compounds, and higher concentration of total procyanidins and total flavonoids, compared to flesh [71]. Kalinowska et al. [72] demonstrated that apple peel from the ‘Gold Millennium’ apple contains five times the concentration of phenolic compounds compared to the flesh [68]. Similarly, the peel of 15 different apple cultivars had greater concentration of all phenolic compounds compared to the apple’s flesh [73]. In general, evidence from multiple studies comparing various cultivars of apples has shown that apple peel contains all groups of phenolic compounds and has greater concentration of procyanidins and total flavonoids compared to the flesh [59,74,75,76]. However, there are some exceptions. Procyanidin B1 concentration in the flesh of ‘Gloster’, ‘Elstar’, and ‘Gala’ is higher compared to their peel [59] and apple flesh of ‘Lodel’ is higher in phloridzin than its peel [77]. Quercetin glycosides are usually found only in the peel [73]. On the other hand, chlorogenic acid can be found in both the flesh and the peel of ‘Golden Delicious’, ‘Granny Smith’, and ‘Idared’ apples, but tends to be higher in the flesh [59,69]. These findings are in accordance with the study from Kschnosek et al. [73], where the predominant group found in the flesh was the phenolic acids (43%), while the flavonols, namely quercetin and its glycosides, were enriched in the peel (72%) and not detected in the flesh. Taken together, these data suggest that apple peel of most apple varieties contain more phenolic compounds than the flesh.

It is important to consider that apple peel contributes only up to 10% of the weight of the whole fruit; therefore, the intake of some phenolic compounds from the peel after consumption of a whole apple might not be as significant as the intake from the flesh. Only a few studies have reported on the phenolic compounds content relative to the weight of the peel compared to the whole apple [62,71]. McGhie et al. [80] demonstrated that peel of ‘Braeburn’, ‘Royal Gala’, and ‘Red Delicious’ contributed 55%, 50%, and 52%, respectively, of the apple’s total phenolic compounds. Similarly, apple peel of ‘Granny Smith’, ‘Idared’, ‘Red Rome’, ‘Jonmac’, ‘Gloster’, and ‘Starking Delicious’ contributed 50% or more to the apple’s total phenolic compounds content. In contrast, the peel of ‘Pilot’, ‘McIntosh’, and ‘Prima’ contributed less to the total phenolic compounds of the whole apple [71]. Data from New Zealand heritage apple cultivar ‘Monty’s Surprise’ and the commercial varieties ‘Braeburn’ and ‘Red Delicious’ (Table 2) showed that the contribution of total phenolics was lower from the peel compared to the flesh. However, anthocyanidins were only present in the peel and flavonols were only found in small quantities in the flesh. Taken together, a combination of unpublished data from New Zealand (Table 2) and other published studies suggests that for most apple varieties the peel is a significant source of phenolic compounds. Therefore, discarding the peel during production of some traditional apple products, such as apple sauce [81], may decrease the health potential of the apples.

The health benefits of an apple’s bioactive compounds depend on their absorption, metabolism, and distribution within the human body [82]. The bioavailability of phenolic compounds (the fraction of the bioactive that has been absorbed and is available for biological activity) is affected by pH, enzymatic activity, their chemical structure, solubility, free and bound form, and synergistic effects with the food matrix (see Section 5.2) [83,84,85].

Despite absorption of phenolic compounds beginning in the small intestine [85], most of the compounds are released in and absorbed from the large intestine with aid from the gut microbiota [84,86,87]. The gut microbiota is capable of transforming complex phenolic compounds into metabolites that are more easily absorbed [88]. It was demonstrated that once absorbed, phenolic compounds can be detected in human plasma and urine after consumption of apple [89], apple juice [90], and apple cider [91]. Bioavailability of the main apple phytochemicals is described in Section 5.2.

While apple is a rich source of nutrients and phytochemicals, there is evidence to suggest that the apple food matrix (non-nutrient component) plays an important role in the absorption and bioavailability of apple phytochemicals. Aprikain et al. [87] demonstrated that the ingestion of phenolics-rich apple extract and apple fiber (pectin) together had greater effect on gut microbiota metabolism in the large intestine and lipid metabolism than ingestion of the phenolics-rich apple extract alone, suggesting a beneficial interaction between fiber and phenolic compounds [87]. Recent studies have shown that a whole apple has strong prebiotic effects [92,93] and that its fiber content promotes the bioaccessibility of other beneficial phytochemicals [94,95]. Additionally, dietary fiber fermentation by the gut microbiota releases short chain fatty acids that have been shown to modulate expression of cell cycle-regulating proteins and induce apoptosis in colon cancer cells [96]. The majority of dietary fiber originates in the plant cell wall and many phytochemicals are known to bind plant cell wall components [94]. As such, processing of apples (including juicing and cooking) will influence the plant cell wall integrity and fiber content of the fruit, which will consequently change the phytochemical’s bioaccessibility, bioavailabilty, and interactions with the gut microbiota. However, processing and ultra-processing techniques are diverse and complex and their impact on health is outside the scope of this review. The influence of the food matrix (mainly fiber and carbohydrates) on the main apple phytochemicals is described in Section 5.2. 

The studies reviewed in this section highlight that there are large variabilities in the composition of phytochemicals in apples and that the phytochemical patterns and profiles can vary in relation to cultivar and apple part. Therefore, the type and level of health benefit will vary in relation to the phytochemical profile of the apple consumed. Ultimately, the phytochemical compounds from apple will only achieve benefit once they become bioavailable and reach the cells and tissue of interest.

## 5. Health Benefits of Apple Phytochemicals: Cancer

Current research attributes the health benefits of apples mainly to the phenolic compounds which exhibit several biological functions beneficial for human health [54]. Apple phenolic compounds are believed to lower incidence of chronic conditions such as cardiovascular disease, cancer, asthma and pulmonary disease, diabetes, and obesity [49,97,98,99,100,101,102,103].

**Figure 3:**
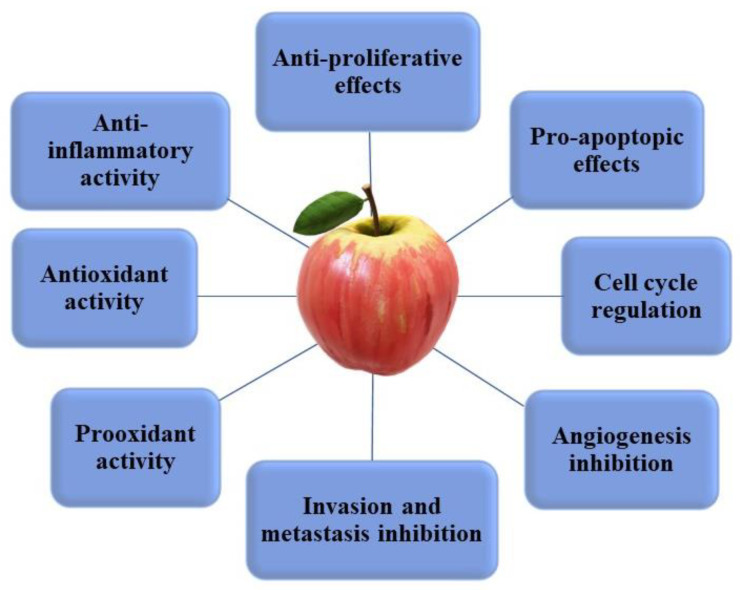
Main mechanisms of action of apple phytochemicals on cancer cells.

Apple phytochemicals are suggested to have many chemo-preventive and chemo-protective effects (Figure 3) against various types of cancer. These effects include regulation of proliferation, cell cycle, apoptosis, reactive oxygen species (ROS), and anti-inflammatory activities [36,47,86,104,105]. In this section, we discuss the health benefits of apple phytochemicals in relation to cancer from epidemiological studies, their ability to alter ROS in cancer cells, and impact on cancer biology from in vitro and in vivo studies. 

### 5.1. Epidemiological Evidence of Apple Consumption and Cancer Incidence

Epidemiological studies have associated apple and pear consumption with lower incidence of different cancers. Reports from the European Prospective Investigation into Cancer and Nutrition (EPIC) cohort study demonstrated that consumption of apples and pears is associated with lower lung [106] and bladder [107] cancer incidence. Consumption of apples and pears was also associated with lower lung cancer incidence in the American Nurses’ Health Study [108]. Apple consumption in particular was associated with lower lung cancer incidence in epidemiological studies from [109], Hawaii [110], and the Zutphen elderly study (Netherlands) [111]. Furthermore, consumption of apples and pears has been associated with lower breast cancer risk from a pooled analysis of two large prospective studies (Nurses’ Health Study—NHS and NHSII) [112]. In one case-control trial from Italy, it was reported that consumption of more than three apples or pears a day was inversely related to pancreatic cancer [113], which is an extra fruit portion above the dietary recommendation by WHO. Both apples and pears are rich in polyphenols, are popular fruit, and are widely available all year round in many countries. Therefore, it is not surprising that apples and pears are identified together in many observational studies that assess dietary habits.

Apple consumption specifically was associated with lower cancer incidence in several observational studies. Apple consumption was associated with decreased breast cancer incidence in a fruit and vegetable study conducted on pooled cohorts [114] and a case-control study from Mexico in pre-menopausal women [115]. Similarly, consumption of apples was associated with reduced incidence of colorectal [116], oral cavity and pharynx [47,117], esophagus [47], larynx [47], ovary [47], renal [118,119], and prostate [47,120] cancers. One case-control study looking at fruit and vegetable consumption in pre-menopausal women in Shanghai showed an inverse association with fruit intake and breast cancer [121]. While the study found the strongest association was with citrus fruit, consumption of 57 g/day of apple or more was also reported to reduce incidence of breast cancer in the study [121]. In a meta-analysis of 20 case-control studies and 21 cohort studies, it was shown that apple consumption was associated with a reduced risk of lung, colorectal, oral cavity, and breast cancers [122].

Findings from the epidemiological studies reported in this review suggest that the consumption of apples reduce cancer risk. However, the cohort size and composition as well as the intervention vary between the different studies. Additionally, other dietary and lifestyle factors could influence cancer outcome in these cohort and observational studies. Further studies are needed to specifically clarify the effect of apple consumption on the incidence of cancer. In addition, most of these studies are observational and to date, there are no clinical intervention trials reported demonstrating the link between apple consumption and cancer incidence. Further research and clinical studies would help to better understand and confirm the effect of apple phytochemicals on cancer in humans.

### 5.2. In Vitro and In Vivo Evidence of the Anticancer Properties of Apple Phytochemicals

Apple phytochemicals were reported to have significant effects on inhibiting multiple ‘hallmarks of cancer’ (detailed below) which are important in the progression of cancer [123].

Phenolic compounds from different apple cultivars were positively associated with the higher degree of inhibition of breast cancer cell proliferation [124,125,126] and induction of cell cycle arrest [125,127]. Additionally, apple extracts inhibited growth of prostate [127] and lung [128] cancer cells. Extracts of phenolics from apple pomace of different apple cultivars were reported to inhibit proliferation of oral [129] and colon cancer cells [58]. In addition to in vitro studies, apple polyphenol extracts also inhibited ex vivo proliferation of a hepatoma cell line [130].

Multiple studies have demonstrated that apple phytochemicals can inhibit the activity of p21, growth factors, pyruvate dehydrogenase kinases (PDKs), cyclin-dependent kinases (CDKs), and extracellular protein kinases (ERKs) essential for cell cycle progression [37,126,131,132]. Furthermore, apple phytochemicals can also prevent cell cycle progression by activation of maspin, a tumor suppressor gene [127]. Reduced expression of the key molecules essential for regulating cell cycle such as phosphorylated Rb, Cyclin D1, and CDK4 by apple phytochemicals lead to cancer cells’ arrest [125,127]. Apple extracts were reported to inhibit apoptosis in breast cancer cells [124,128]. Apple phenolic compounds were shown to elevate the expression of pro-apoptotic genes such as p53 and Bax and reduction in the expression of anti-apoptotic genes such as p21 and Bcl-2 [133]. In addition to inhibiting cell proliferation and promoting apoptosis, apple phytochemicals have also been implicated in inhibiting angiogenesis by regulating VEGF [123] and inhibiting invasion and metastasis [58,130] by regulating matrix metalloproteinases-2,-9 (MMP-2,-9), cadherins and integrins [123], and regulating COX-2 a marker of inflammation [123]. Additionally, the ability of apple phytochemicals to inhibit cell proliferation and in turn reduce incidence of cancer was also observed in rats fed with one human apple equivalent. These rats had reduced appearance of different precancerous markers (ACF, MDF, genes, and proteins related to colorectal cancer progression) [134]. Similarly, the incidence of mammary tumors in rats was reduced after two weeks of oral administration of 3.3, 10, and 20 g of apple extract/kg of body weight, which correspond to the human consumption of one (200 g), three, and six apples per day, respectively [135].

There is evidence that anticancer properties of apples are due to the synergistic effects between apple phytochemicals and the food matrix [136,137,138,139]. Veeriah et al. [136] demonstrated that colon cancer cells treated with an apple extract (extract from a mixture of different apples) reduced cell proliferation to a greater extent compared to a synthetic apple extract composed of eight apple phenolic compounds or individual apple phenolic compounds. They further demonstrated that colon cancer cells treated with a synthetic apple extract composed of eight apple phenolic compounds also reduced cell proliferation to a greater extent compared to individual apple phenolic compounds. [136]. Results from this study indicate the importance of the apple food matrix, which may contain other bioactive compounds present in the apple extract but not in the synthetic mixture. 

Taken together, evidence from in vitro, ex vivo, and in vivo studies suggest that apple phytochemicals work synergistically to inhibit multiple ‘hallmarks of cancer’, which in turn can influence cancer incidence and improve outcomes to chemotherapeutic treatments. 

Oxidative stress can result in direct or indirect ROS-mediated damage of macromolecules such as DNA, proteins, and lipids, allowing cells to acquire multiple ‘hallmarks of cancer’ and facilitating carcinogenesis [55,140,141]. Phenolic compounds such as quercetin, epicatechin, procyanidin B2, phloretin, and chlorogenic acid were identified as the biggest contributors to the apple’s antioxidant activity [142]. In one study, apples showed the second highest antioxidant activity in vitro after cranberries among 11 common fruits tested [57]. Both apple peel and flesh extracts from dried and lyophilized apples of four different cultivars reduced ROS in the lipopolysaccharide (LPS)-induced mouse brain microglia cells (BV-2), with apple peel having a greater antioxidant effect [143]. While these studies have indicated the antioxidant capacity of apples, it is important to note that the methods used to measure antioxidant activity (such as total antioxidant capacity) are synthetic assays and do not necessarily capture the complexity of a physiological system [144]. Nevertheless, it has been proposed that antioxidant activity of apple phytochemicals can inhibit or reduce cancer cell proliferation [124,128,129,145,146,147] and promote apoptosis [148] of cancer cells based on in vitro studies (Table 3). 

On the other hand, many bioactive compounds can work as prooxidants and under certain conditions (high concentration, presence of metal ions, and low pH) can induce ROS production and promote cell death [149,150,151]. In cancer therapy, prooxidants may have a beneficial effect by working as cytotoxic agents for fast growing cells and inducing cancer cell death [152,153,154]. Prooxidant activity of many phenolic compounds has been associated with their ability to induce apoptosis and cell cycle arrest in different cancer cells [155,156,157]. For example, Mendoza-Wilson et al. [158] demonstrated that phloridzin exhibited prooxidant activity [158]. Catechin and epicatechin are well known antioxidants, however, they can act also as prooxidants [159,160]. Epicatechin induced ROS production in colon cancer cells led to activation of pro-apoptotic enzymes and therefore induced apoptosis of these cells [159]. 

These examples suggest that some phytochemicals have a biphasic or hormetic response depending on the dose administered [161]. Therefore, phytochemicals such as the ones present in apple can be used to produce an antioxidant effect for cancer prevention, but also induce prooxidant effects with benefits in cancer prevention. 

In the subsequent sections. the review will focus on the anticancer mechanisms of the main apple phenolic compounds (quercetin, phloretin, chlorogenic acid, catechins, epicatechins, and procyanidins) and triterpenoids. The data on in vitro activity of apple phenolics with effective concentrations are summarized in Table 3. 

#### 5.1.1. Quercetin Anticancer Properties

Of all the apple phenolic compounds, quercetin glycosides are the most efficiently absorbed compounds from apples [162,163,164], mainly absorbed in the large intestine. Quercetin in apples is present in glycoside forms, and interestingly, these are more readily absorbed compared to quercetins from tea [165] but less readily absorbed compared to the quercetins from onion [166]. Quercetin glycosides can be absorbed as intact molecules where the sugar moiety helps to improve absorption through the gut lumen [167]. Furthermore, the presence of carbohydrates and pectin in the apple food matrix significantly increases quercetin absorption [168,169,170,171]. Quercetin has been detected in the plasma of humans (Cmax 0.30 μM/92 ng/mL, Tmax 2.5 h) [166] and rats (Cmax 118 μM ± 08) [172] after apple consumption. These studies suggest that quercetin is absorbed and present in the plasma at a sufficient concentration to elicit the anticancer effects. 

Recent in vitro and in vivo studies have reported that quercetin is one of the flavonoids responsible for the apple’s anticancer properties [173,174]. Quercetin has been shown to inhibit cell proliferation of breast [175,176,177,178], ovarian [179], lung [180,181], and liver [180] cancer cells in vitro (Table 3). Additionally, studies have demonstrated quercetin’s ability to induce apoptosis in multiple cancer cell lines ([177,178,182,183], Table 3) and in vivo using colon cancer cell line xenografts in mice [182]. The ability of quercetin to promote apoptosis and cell cycle arrest is believed to be due to its regulation of p53, GADD45, and AMPK [182,184]. Autophagy is another form of cell death in which damaged organelles are degraded [185]. Interestingly, quercetin is also shown to induce autophagy in lung cancer cells [181] and in breast cancer bearing mice by reducing the activity of AKT-mTOR pathway [186]. Additionally, quercetin inhibited invasion and migration of breast [186] and colorectal [187] cancer cells (Table 3). Quercetin was also shown to inhibit the activity of VEGFR2 and therefore inhibit angiogenesis in breast [188], prostate [189], and retinoblastoma [190] cancer cells. 

Based on the results from in vitro studies, quercetin has strong anticancer properties in different cancer cells. However, the absorption and metabolism of quercetin glycosides from whole foods such as apples are not well known. Therefore, to better understand the anticancer effects of quercetin in humans, further studies are needed to identify factors that influence quercetin mechanisms of action and bioavailability in vivo.

#### 5.1.2. Phloretin and Phloridzin Anticancer Properties

Phloretin and its glucoside form (phloridzin) are other flavonoids found in apple. Phloretin and phloridzin are metabolized into phloretin glucuronides and phloretin sulfate glucuronides in the human intestine [169]. Phloridzin has been detected in the plasma (Cmax 66.9.0 μM ± 19.4, Tmax 10 h) and urine of rats [191,192]. It has also been detected in the plasma of humans (Cmax 73 nM ± 11, Tmax 0.6 h) [193]. Phloretin and its glycosides has been detected in ileal fluid in humans after consumption of apple juice [194] and cider [193], whereas phloridzin was not detected, suggesting that phloridzin is more readily absorbed. 

Based on the available evidence, it is unclear how the apple food matrix and composition of the gut microbiota would affect uptake and metabolism of phloretin, which in turn may impact its anticancer properties against cancer cells. Despite limited information on the bioavailability of phloretin, several lines of evidence support its anticancer properties from in vitro and in vivo studies. Phloretin was shown to inhibit proliferation of breast cancer [195] and colorectal [196] cancer cell lines via inhibition of glucose transporter 2 (Glut-2) in vitro and in vivo [197]. Additionally, phloretin inhibited proliferation by promoting cell cycle arrest [198,199], ROS production [200], apoptosis [198,201,202,203], and by inhibition of autophagy via mTOR/ULK1 [204]. Phloretin also inhibited invasion and migration [197,200,205,206]. Moreover, phloretin promoted an anti-inflammatory environment by inhibiting the expression of pro-inflammatory molecules PGE2, IL-8 and advanced glycation end products (AGEs) receptor [207]. Finally, evidence from in vivo studies suggest that phloretin may enhance the effect of commercial chemotherapeutic drugs such as Paclitaxel [208]. 

Details of these in vitro and in vivo studies are provided in Table 3. It is important to note that most of the in vitro cancer studies use synthetic phloretin, thus, to maximize the anticancer benefits of phloretin, there is a need to further our understanding of phloretin bioavailability.

#### 5.1.3. Chlorogenic Acid Anticancer Properties

Chlorogenic acid is one of the phenolic acids abundant in apples and is reported to have anticancer potential, details of which have been summarized in Table 3. Chlorogenic acid is metabolized mainly in the large intestine by the gut microbiota from its aglycone form to its microbial metabolites, some of which include caffeic acid, 3-phenylpropionic acid, 3-phenylpropionic acid, hippuric acid, and quinic acid [209,210,211,212]. Interestingly, after consumption of foods rich in chlorogenic acid, its microbial products have been detected in the plasma of rats (Cmax 0.34 μM, Tmax 1 h) and human urine [209,210,213] but not its aglycone forms [209,210]. Studies in vitro and in vivo have demonstrated that chlorogenic can inhibit cell proliferation [214,215,216,217,218], promote cell cycle arrest [214,218,219] via affecting miR-17 family [214], induce apoptosis [26,218] by binding to annexin and suppressing the NF-κB pathway [220,221], and inhibit invasion and metastasis via downregulation of MMP-2 and MMP-9 [216]. Chlorogenic acid also inhibited growth of the liver [216] and breast [220,221] cancer tumor in xenograft mice in vivo.

Chlorogenic acid possesses different anticancer properties in vitro. However, the limiting factor for its anticancer properties in humans is its low bioavailability. The microbial produced metabolites of the gut might be contributors to the chemo-preventive effects of chlorogenic acid, however, further research in this area is needed.

#### 5.1.4. Catechins and Epicatechins Anticancer Properties

Catechins and epicatechins are monomeric flavanols that are unstable in the gastrointestinal tract and poorly absorbed [222], with only 1.6% of the ingested catechins/epicatechins from tea, detected in human plasma, feces, and urine (plasma epicatechin Cmax 174 nM, Tmax 7 h) [223]. Similar results were obtained in the plasma of rats where the oral bioavailability of radioactively labeled catechin was about 3% and for radioactively labeled epicatechins was 4% of the orally administrated dose (catechins: Cmax 30.40 ± 1.80 ng/mL, Tmax 1.25 h; epicatechins: Cmax 196.5 ± 18.1 ng/mL, Tmax 1.1 h) [224,225]. Notwithstanding the limitation with their bioavailability, catechins and epicatechins have been reported to have a multitude of anticancer properties summarized in Table 3. Both catechins and epicatechins in vitro and in vivo have inhibited cancer cell proliferation [226,227,228], induced cell cycle arrest by upregulating the expression of p21 [229] and inhibition of CDC25A [226], induced apoptosis [227,229,230,231,232,233], and reduced invasion and migration [233]. 

In several of these studies, the catechins and epicatechins used were derived from sources other than apple. However, the impact of the apple matrix and the gut microbiome on the bioavailability of catechins and epicatechins remains unknown. Thus, further studies are required to investigate the bioavailability and anticancer properties of epicatechins and catechin from apples.

#### 5.1.5. Procyanidins Anticancer Properties

Procyanidins are another group of oligomeric flavanols, which are abundant in apples and have many anticancer properties. Procyanidin dimers from grape seed extract and cocoa were detected in human plasma (Cmax 16 ± 5 nM, Tmax 0.5 h; Cmax 10.6 ± 2.5 nM, Tmax 2 h) [234,235] but not rat plasma [236]. Moreover, larger procyanidins were not absorbed efficiently by intestinal epithelial cells and are metabolized to low weight phenolic acids by the gut microbiota [237]. It was reported that compared to other apple phenolics, procyanidins have a greater effect on cancer cell proliferation and apoptosis in vitro [238,239]. Like their monomeric counterparts (catechins and epicatechins), procyanidins have been shown in vitro and in vivo to inhibit proliferation [238,240,241,242], induce cell cycle arrest [238,240,241], promote apoptosis [238,239,243,244], induce ROS [245,246], inhibit migration [242], and angiogenesis [247]. In addition, procyanidins isolated from apple also inhibited breast [239] and liver [242] cancer tumor growth in xenograft mouse. Table 3 provides details of the in vitro studies. 

The bioavailability of procyanidins varies and is dependent on their structure and the composition of the gut microbiome, where the metabolites may elicit the anticancer effects. Therefore, to further understand the anticancer properties of procyanidins, studies are needed to investigate the anticancer properties of their metabolites and their bioavailability in humans.

#### 5.1.6. Triterpenoids Anticancer Properties

Triterpenoids are another group of bioactive compounds found predominantly in apple peel. Triterpenoids are suggested to contribute to anticancer activity, details of which are summarized in Table 3. Triterpenoids consist of oleanolic acid and its isomer ursolic acid and betulinic acid. Triterpenoids are known to have low bioavailability and are poorly absorbed in the intestine [248,249]. It has been shown that only 2.3% of orally administered betulinic acid was detected in mouse plasma (Cmax 3.1 μg/mL, Tmax 2 h) [250], while only 0.7% of orally administered oleanolic acid was detected in rat plasma samples (Cmax 74.0 ± 57.2 ng/mL, Tmax 25 mins) [251]. In contrast, oleanolic acid has been detected in human plasma four hours after raisin consumption (Cmax 24.4 ± 14.4 ng/mL, Tmax 4 h) [252]. Like other apple phytochemicals, triterpenoids were shown to inhibit proliferation [253,254,255,256,257,258,259,260], induce apoptosis [80,253,254,255,257,258,259,261,262,263,264,265,266,267], alter ROS production [262,268,269], inhibit invasion and metastasis [255,256,257,270,271,272,273], and angiogenesis [270]. 

Current research on apple phytochemicals is focused more on the anticancer properties of apple phenolic compounds with relatively few studies looking at apple triterpenoids. Future research on other triterpenoids and their derivatives present in apples such as pomaceic acid (which is unique to the apples) or pomolic, euscaphic, and maslinic acid is necessary to better understand the anticancer properties specific to apples.

**Table 3:** Summary of mentioned in vitro studies with the effective concentration.

In Vitro	Effect	Expression Markers Affected	Effective Concentration	Cell Line	Ref.
QUERCETIN	Lung cancer				
	Anti-proliferative	↓ PDK3	55.90 ± 2.25 µM	A549	[180]
	Anti-proliferative, pro-apoptotic, autophagy inhibition	↑LC3-II, SIRT 1, AMPK, beclin 1 ↓ p62	100 µM	A549, H1299	[181]
	Breast cancer				
	Anti-proliferative, cell cycle arrest	↑cyclin B1 and CDK-1 ↓p21	10 µM	SK-BR3, MDA-MB-453	[175]
	Anti-proliferative, pro-apoptotic	↑ miR-146a, bax, caspase-3 ↓ EGFR	80 µM/mL, 50 µM/mL (respectively)	MCF-7, MDA-MB-231	[177]
	Anti-proliferative, pro-apoptotic	↓ survivin	40 mg/mL	MCF-7	[178]
	Pro-apoptotic, cell cycle arrest	↓ Foxo3a, p53, GADD45	20 µM	MDA-MB-231	[182]
	Metastasis and invasion inhibition	↓ MMP-2,9, VEGF, PKM2, GLUT1, LDHA, Akt, mTOR	30 µM	MCF-7, MDA-MB-231	[186]
	Angiogenesis inhibition	↓ VEGF, Pin1	30 µM	MCF-7	[188]
	Colorectal cancer				
	Metastasis and invasion inhibition, anti-inflammatory	↑ E-cadherin ↓ MMP-2,9, p65, TLR4, TNF-α, COX-2, IL-6	5,10, 20 μM	Caco-2	[187]
	Liver cancer				
	Anti-proliferative	↓ PDK3	49.10 ± 1.45 µM	HepG2	[180]
	Ovarian cancer				
	Pro-apoptotic	↑ phospho-eIF2α, p53 ↓ Rad51	25, 50, 75, 100 µM	OV2008, A2780, GM9607	[183]
	Anti-proliferative, pro-apoptotic, cell cycle arrest	↓ survivin	30 mg/ml	SKOV-3	[179]
	Prostate cancer				
	Anti-proliferative, angiogenesis inhibition	↓ Akt, mTOR, VEGFR2, S6 kinase	10-40 mmol/L	HUVECs	[189]
	Retinoblastoma				
	Angiogenesis inhibition	↓ VEGFR	25, 50, 200 µM	Y79	[190]
PHLORETIN AND PHLORIDZIN	Lung cancer				
	Anti-proliferative, pro-apoptotic, invasion and migration inhibition	↑ caspase-3,-9 ↓ Bcl-2, MMP-2,-9	25, 50, 75 µg/mL	A549, H838, H520, Calu-1	[205]
	Pro-apoptotic, cell cycle arrest	↑ Bax, caspase-3, -9 ↓ Bcl-2	50, 100, 200 µM	A549	[199]
	Breast cancer				
	Anti-proliferative, cell cycle arrest	↓ GLUT-2	25, 50, 100, 150 µM	MDA-MB-231	[195]
	Anti-proliferative, autophagy inhibition	↓ mTOR, ULK1, LC3B-II	100, 200 µM	MDA-MB-231, MCF7, ERα+	[204]
	Colorectal cancer				
	Anti-proliferative, cell cycle arrest	↑ E-cadherin, p53 ↓ GLUT-2	100, 200 µM	Colo 205, HT-29	[196]
	Pro-apoptotic	↑ caspase-3,-7, -9, Bax, cytochrome C ↓ Bcl-2	100 μmol/L	HT-29	[206]
	Anti-inflammatory	↓ PGE2, IL-8, AGEs	50 μM	CCD-18Co	[207]
	Liver cancer				
	Pro-apoptotic, invasion and migration inhibition	↓ GLUT-2, Bcl-2, Akt	200 μM	HepG2	[197]
	Pro-apoptotic	↑ SHP-1 ↓ p-Akt, pERK, mTOR, VEGFR2, p-JNK	25, 50, 100 μM	SK-Hep1, Hep3B2.1-7, Huh7, PLC5, HepG2	[201]
	Prostate cancer				
	Prooxidant, anti-proliferative, migration inhibition	↑ ROS ↓ β-catenin, TCF4, FoxA2, c-Myc, CISD2	20, 50, 100 μM	PC3, DU145	[200]
	Gastric cancer				
	Anti-proliferative, pro-apoptotic, cell cycle arrest	↓ p-JNK, p-38	4, 8, 16 μM	AGS	[198]
	Esophageal cancer				
	Anti-proliferative, pro-apoptotic	↑ BAX, p53 ↓ Bcl-2	60, 70, 80, 90, 100 μg/mL	EC-109	[203]
	Brain cancer				
	Anti-proliferative, pro-apoptotic, cell cycle arrest	↑ p27 ↓ CDK-2,-4,-6, cyclin-D,-E	100, 200, 300 μM	U87, U251	[202]
CHLOROGENIC ACID	Lung cancer				
	Anti-proliferative, cell cycle arrest	↑ p21, p53, and KHSRP ↓ c-Myc, miR-17 family	25, 50 μM	H446	[214]
	Anti-proliferative, pro-apoptotic	↓ cIAP1, cIAP2, binding of annexin A2 to p50 and actin => ↓ NF-κB	25, 50, 100, 200, 400, 800 μM	A549	[220]
	Anti-proliferative, pro-apoptotic	↑ Bax, caspase-3, p38, JNK, annexin V ↓ Bcl-2, SOX2	30, 50 μM	A549	[215]
	Breast cancer				
	Anti-proliferative, pro-apoptotic, migration and invasion inhibition	↓ annexin, NF-κB, p65	10,20 μM	MDA-MB-231, MDA-MB-453	[221]
	Colorectal cancer				
	Anti-proliferative, cell cycle arrest, prooxidant	↑ ROS, p53 ↓ ERK	125, 250, 500, 1000 μmol/L	HCT116, HT29	[217]
	Cell cycle arrest, pro-apoptotic	↑ caspase-3	250, 500, 1000 μM	Caco-2	[219]
	Liver cancer				
	Anti-proliferative, cell cycle arrest, invasion, and metastasis inhibition	↓ MMP-2,-9, ERK1/2	250, 500, 1000 μM	HepG2	[216]
	Anti-proliferative, cell cycle arrest	↑ p21, p53, and KHSRP ↓ c-Myc, miR-17 family	25, 50 μM	Huh7	[214]
	Kidney cancer				
	Anti-proliferative, pro-apoptotic	↑ caspase, Bax ↓ Bcl-2, PI3K, Akt, mTOR	40 μM	A498	[26]
	Osteosarcoma				
	Anti-proliferative, pro-apoptotic, cell cycle arrest	↑ ERK1/2	200, 400 μM	U2OS, Saos-2	[218]
CATECHIN AND EPICATECHIN	Breast cancer				
	Pro-apoptotic	↑ ZIP9 ↓ cAMP agonists to membrane androgen receptors	200 nM	MDA-MB-468	[230]
	Pro-apoptotic	agonists to membrane androgen receptors	21.4 nM	T47D	[231]
	Anti-proliferative, pro-apoptotic, antioxidant	↑ IRK ↓ ROS	40, 100 μg/mL	MCF-10A	[227]
	Pro-apoptotic, prooxidant	↑ ROS, Bad, Bax	150, 200, 250, 300, 350, 400, 450, 500 μM	MDA-MB-231	[232]
	Colorectal cancer				
	Pro-apoptotic, migration and invasion reduction	↑ E-cadherin ↓ ERK1/2, c-Myc, β-catenin	12.5, 20 μM	HT-29	[233]
	Liver cancer				
	Anti-proliferative, cell cycle arrest	↑ p21, waf1/cip1 ↓ CDC25A	50, 75, 100, 125, 150 μM	HepG2, Huh7	[226]
	Biliary tract cancer				
	Pro-apoptotic, cell cycle arrest	↑ caspase, p21, gene dr5	20, 50 μM	CCSW-1, BDC, EGI-1, SkChA-1, TFK-1, MzChA-1, MzChA-2, GBC	[229]
	Prostate cancer				
	Pro-apoptotic	↑ ZIP9 ↓ cAMP agonists to membrane androgen receptors	200 nM	PC-3	[230]
	Pancreatic cancer				
	Anti-proliferative, pro-apoptotic, cell cycle arrest	↑ Bax ↓ Ras, NF-κB, p65, Bcl-2, Pi3K, Akt	25, 50 μM	E6E7-Kras-st	[228]
PROCYANIDINS	Breast cancer				
	Pro-apoptotic	↑ cytochrome-c, caspase-3,-9	25 μg/m	B16, BALB-MC.E12	[239]
	Pro-apoptotic, migration and invasion reduction	↑ maspin, E-cadherin, BRCA1 ↓ DNA methyltransferases	50, 100, 150, 200, 250 μM	MDA-MB-231	[243]
	Pro-apoptotic	n/a	50 μM	MCF-7	[244]
	Pro-apoptotic, cell cycle arrest	↑ Bax, caspase-3,-9 ↓ Bcl-2	31.5, 36.6 mg/mL	MDA-MB-231, MCF-7	[274]
	Colorectal cancer				
	Anti-proliferative, pro-apoptotic, cell cycle arrest	↑ caspase-3, ERK1/2, JNK ↓ PKC	45 μg/mL	SW620	[238]
	Anti-proliferative, pro-apoptotic, cell cycle arrest	↑ MMP-2,-9, caspase-3,-9, ERK 1/2, MEK, Akt, PI3K ↓ EGFR	10–60 μM	Caco-2, HT-29, HCT-15, HCT-116	[240]
	Pro-apoptotic, prooxidant	↑ caspase-3,-8,-9, Bax, ROS, cytochrome-c ↓Bcl-2	80 µg/mL	SW480 and SW620	[246]
	Anti-proliferative, cell cycle arrest, pro-apoptotic	↑ ERK1/2, MEK, PI3K, Akt ↓ EGFR	10, 20, 30 μM	Caco-2	[241]
	Pro-apoptotic	↑ PKB, Akt, ERK1/2, p38	2.5–20 μM	Caco-2	[275]
	Pro-apoptotic	↑ caspase-3,-9, cytochrome-c ↓ PI3K, Akt, bad	2.5–50 μM	Caco-2	[276]
	Liver cancer				
	Anti-proliferative, migration inhibition	↓ Kv10.1	10, 100, 1000 μM	HepG2	[242]
	Prostate cancer				
	Pro-apoptotic, prooxidant	↑ ROS, ERK1/2, AMPKα	25, 50 μM (PCa LNCaP); 50, 100, 200 μM (22Rv1)	PCa LNCaP, 22Rv1	[245]
TRITERPENOIDS	Breast cancer				
	Anti-proliferative	n/a	n/a	MCF-7	[260]
	Anti-proliferative, pro-apoptotic	↑ Bax, cytochrome-c, p53 ↓ Bcl-2	2.57, 5.45 μM (respectively)	MDA-MB-231, MCF-7	[254]
	Anti-proliferative, migration and invasion inhibition	↑ caspase-3 ↓ MMP-2,-9, TIMP-2	5, 10, 20 μM	MCF-7, 4T1, MDA-MB-231	[256]
	Anti-proliferative, pro-apoptotic, migration and invasion inhibition	↓ aerobic glycolysis, c-Myc, lactate dehydrogenase A (LDH-A), p-PDK1, Caveloin-1	48.55, 19.06 μM (respectively)	MDA-MB-231, MCF-7	[255]
	Anti-proliferative, pro-apoptotic, migration and invasion inhibition	↑ GRP78, PERK ↓ aerobic glycolysis, c-Myc, β-catenin	5-50 μM	MDA-MB-231, BT-549, HBL-100	[257]
	Anti-proliferative, pro-apoptotic, autophagy inhibition, anti-inflammatory	↓ PI3K, Akt, NF-κB	232, 221, 240 μg/mL (respectively)	T47D, MCF-7, MDA-MB-231	[258]
	Cell cycle arrest, pro-apoptotic, autophagy	↑ p53, p21, AMPK ↓ ERK1/2, glycolysis, PKM2, HK2	20 μM	MCF-7, MDA-MB-231, SK-BR-3	[80]
	Lung cancer				
	Pro-apoptotic, angiogenesis inhibition	↑ Bax ↓ VEGF	25, 50 μg/ml	A549, H460	[270]
	Colorectal cancer				
	Anti-proliferative	n/a	n/a	Caco-2	[260]
	Invasion and metastasis inhibition	↓ cadherins, integrins	10, 20, 40, 80 μM	SW620	[272]
	Liver cancer				
	Anti-proliferative, prooxidant	↑ ROS ↓ PI3K, Akt1, mTOR	10, 30, 100 μM		[268]
	Anti-proliferative	n/a	n/a	HepG2	[260]
	Invasion and metastasis inhibition	↓ cadherins, integrins	10, 20, 40, 80 μM	HepG2	[272]
	Anti-proliferative, pro-apoptotic	↑ p53, caspase-3 ↓ Bcl-2, Mcl-a mRNA	10, 20, 30 μM	HUH7, PLC/PRF/5, L02	[265]
	Pancreatic cancer				
	Cell cycle arrest, pro-apoptotic, autophagy induction	↑ Bax, ATG5, LC3-II ↓ Bcl-2, RAGE	25, 50, 75, 100 μM	MIA Paca-2	[266]
	Prostate cancer				
	Pro-apoptotic	↑ cytochrome-c, PARP, p21, p53 ↓ NF-κB, Bcl-2, p65	10, 25 μM	LNCaP, DU145	[263]
	Anti-proliferative, pro-apoptotic	↑ survivin ↓ Bcl-2, Bcl-xl, survivin, PI3K, Akt, mTOR		LNCaP, PC-3	[259]
	Cervical cancer				
	Pro-apoptotic, cell cycle arrest, prooxidant	↑ ROS, p21, Bad, caspase-9 ↓ PI3K, Akt	30 μmol/:	HeLa	[262]
	Ovarian cancer				
	Anti-proliferative, pro-apoptotic	↑ Bax, caspase-3,-8,-9 ↓ Bcl-2	44.47 μM	A2780	[264]
	Gallbladder cancer				
	Anti-proliferative, cell cycle arrest, pro-apoptotic	↑ Bax, cytochrome-c, caspase-3,-9 ↓ Bcl-2	50 μmol/L	GBC-SD, NOZ	[267]
	Brain cancer				
	Pro-apoptotic, migration and invasion reduction	↑ JNK signaling pathway, caspases ↓ enzyme MGMT	20 μM	U373MG	[273]
	Anti-proliferative, pro-apoptotic	↓ enzyme MGMT, STAT3	20, 30, 40, 50 μM	LN229, LN18, T98G	[253]
	Osteosarcoma				
	Anti-proliferative, pro-apoptotic, antioxidant	↑ caspase-3 ↓ Notch signaling pathway, Bcl-2, ROS	50, 80 μM (respectively)	Saos-2, MG63	[269]
	Melanoma				
	Invasion and metastasis inhibition	↓ cadherins, integrins	10, 20, 40, 80 μM	B16-F10	[272]

↑: increased expression; ↓: decreased expression.

## 6. Conclusions

Cancer is a leading cause of death globally and represents one of the greatest health challenges. Therefore, it is necessary to find effective prevention tools to lower incidence of this chronic disease. Phytochemicals are secondary metabolites present in vegetables and fruit that provide many health benefits such as chemo-preventive and chemo-protective effects in different cancers. As highlighted in this review, apples are a promising fruit to consider in dietary plans for cancer prevention as they are widely available and contains various beneficial phytochemicals. There is evidence from epidemiological studies that regular apple consumption decreases the incidence of different cancers. However, from these observational studies, it is difficult to distinguish the effects of apple specifically from other lifestyle factors that influence cancer risk. The anticancer effects of apples are believed to be mainly due to their phenolic compounds such as phloretin, quercetin and its glycosides, chlorogenic acid, catechin, and epicatechin. However, while most of the research is focused on phenolics, there is evidence that triterpenoids, which are present mainly in apple skin, have significant chemo-preventive and chemo-protective effects. A limiting factor of the apple’s anticancer benefits is their low bioavailability in vivo and in humans. 

Apart from the in vivo and in vitro studies that overwhelmingly support the impact of apples in cancer prevention and inhibition, clinical interventions are lacking. Therefore, in this review article, we describe some of the considerations that need to be accounted for when using apples in an interventional setting or dietary plans. The levels and profiles of phytochemicals in apple depends on many factors such as cultivar and maturity stage, but also vary greatly within the apple parts (peel, flesh). Apple’s skin is known to be a rich source of phenolics and significantly contributes to the health benefits of apples, therefore, consumption of whole apple with skin and consumption of different apple cultivars might help to obtain greater anticancer effects. Furthermore, the food matrix components such as fiber have an important role to play in the health benefits of apples and influence the bioavailability of the apple phytochemicals. 

Apple phytochemicals provide many beneficial health effects and could work as a preventive tool in cancer. However, more research (especially in vivo and clinical studies) is needed to confirm apple’s anticancer effects and bioavailability in humans.

## Figures and Tables

**Table 1:** Classification of apple phenolics.

Phenolics Group	Phenolic Subgroup	Phenolic Compounds
Flavonoids	Anthocyanidins	Cyanidin 3-O-arabinoside
		Cyanidin 3-O-galactoside
		Cyanidin 3-O-xyloside
		Cyanidin 3-O-xylgalactoside
	Flavonols	Quercetin
		Quercetin 3-arabinopyranoside
		Quercetin-3-arabinofuranoside
		Quercetin 3-galactoside
		Quercetin 3-glucoside
		Quercetin 3-rhamnoside
		Quercetin 3-rutinoside
		Quercetin 3-xyloside
	Dihydrochalcones	Phloretin
		Phloretin-2′-O-xyloglucoside
		Phloridzin
		3-hydroxyphloridzin
	Flavan-3-ols	Monomeric
		(+)-Catechin
		(−)-Epicatechin
		Oligomeric (Procyanidins)
		Procyanidin B1
		Procyanidin B2
		Procyanidin B5
		Procyanidin B7
		Procyanidin C1
Phenolic acids		Chlorogenic acid Hydroxy benzoic acid Hydroxy cinnamic acid

**Table 2:** Estimated apple peel contribution to the total phenolics content in whole apple. Phenolic compounds were measured using liquid chromatography-mass spectrometry (LC-MS, Dionex Ultimate RS3000 UHPL and a Bruker micrOTOF-QII) in 2019, plant and food research, for 3 apple varieties—Monty’s surprise, Braeburn, and Red Delicious. Each compound concentration was quantified by comparison with an authentic standard where possible or as equivalents to standard compounds. Each phenolic compound in the table is presented as a percentage of total concentration measured using LC-MS. Percentage total phenolics (percentage values presented in bold) was calculated based on the average weight of whole apple (180 g) where apple skin contributed 18 g.

	Monomeric Flavanols		Procyanidins		Flavonols		Dihydrochalcones		Chlorogenic Acid		Anthocyanins		Total Phenolics	
Cultivar	Skin (%)	Flesh (%)	Skin (%)	Flesh (%)	Skin (%)	Flesh (%)	Skin (%)	Flesh (%)	Skin (%)	Flesh (%)	Skin (%)	Flesh (%)	**Skin (%)**	**Flesh (%)**
Monty’ Surprise *	33	67	29	71	94	6	42	58	10	90	100	n.e.	**37**	**63**
Braeburn	19	81	21	79	99	1	8	92	1	99	100	n.e.	**31**	**69**
Red Delicious	37	63	36	64	94	6	40	60	2	98	100	n.e.	**46**	**54**

* Monty’s Surprise—New Zealand’s heritage apple variety. “n.e.”—not evaluated (Anthocyanins were not evaluated in the flesh as they are not present.).

## Abbreviations

AGEs	Advanced glycation end products
Akt	Protein kinase B
AMPK	Activated protein kinase
ATG5	Autophagy protein 5
Bad	Bcl2 Associated Agonist Of Cell Death protein
Bax	Bcl2 Associated X protein
Bcl-2	B-cell lymphoma 2
BRCA1	Breast cancer 1 gene
cAMP	Cyclic adenosine monophosphate
CDC25A	Cell division cycle 25 A gene
CDK-1,-2,-4,-6	Cyclin dependent kinase
cIAP1,2	Cellular Inhibitor of Apoptosis Protein 1
cip1	CDK- interacting protein
CISD2	CISD2 gene
c-Myc	Cellular myelocytomatosis oncogene
COX-2	Cyclooxygenase-2
EGFR	Epidermal growth factor receptor
ERK, ERK1/2	Extracellular regulated kinase
FoxA2	Forkhead box protein A2
FoxO3a	Forkhead box protein o3a
GADD45	Growth arrest and DNA damage-inducible protein
Glut1	Glucose transporter 1
GRP78 (BiP)	Binding immunoglobulin protein
HK2	Hexokinase-2
IL-6,-8	Interleukin-6,-8
IRK	The arabidopsis receptor kinase
JNK	C-Jun N-terminal Kinase
KHSRP	Kh-type splicing regulatory protein
Kv10.1	Kv10.1 potassium channel
LC3-II	Light chain membrane protein
LDHA	Lactate dehydrogenase A
LDH-A	Lactate dehydrogenase A
Mcl-1	Induced myeloid leukemia cell differentiation protein
MEK	Mitogen-activated protein kinase kinase
MGMT	Methylated-DNA—protein-cysteine methyltransferase
miR-146a	Micro Ribonucleic Acid 146a
MMP-2,-9	Matrix metalloproteinase-2,-9
mTOR	Mammalian target of rapamycin protein
NF-κB	Nuclear Factor kappa B
p21, p65, p62, p53, p38, p27	Protein 21,65,62,53,38,27
PARP	Poly [ADP-ribose] polymerase 1
PDK3,1	Pyruvate dehydrogenase
pERK	R-like endoplasmic reticulum kinase
PGE2	Prostaglandin E2
Phosphor-eIF2α	Phosphorylation of eukaryotic initiation factor-2 alpha
PI3K	Phosphoinositide 3-kinase
Pin1	Prolyl isomerase
p-JNK	Phosphorylated c-Jun N-terminal Kinase (JNK)
PKB	Protein kinase B
PKC	Protein kinase C
PKM2	Pyruvate kinase m2
rad51	RAD51 Recombinase (gene)
RAGE	Advanced glycosylation end product-specific receptor
Ras	Ras protein
ROS	Reactive oxygen species
SHP-1	Protein tyrosine phosphatase shp 1
SIRT-1	Sirtiuin 1
SOX2	Sry-box transcription factor 2
STAT3	Signal transducer and activator of transcription 3
TCF4	Transcription factor 4 (gene)
TIMP-2	Tissue Inhibitor of Metalloproteinase 2
TLR4	Toll-like receptor 4
TNF- α	Tumor necrosis factor alpha
ULK-1	Unc-51 like autophagy activating kinase 1
VEGF	Vascular endothelial growth factor
VEGFR	Vascular endothelial growth factor receptor
Waf1	Wild type p53 activated protein-1
ZIP9	Zinc transporter 9
